# Characterization of multinucleated giant cells in synovium and subchondral bone in knee osteoarthritis and rheumatoid arthritis

**DOI:** 10.1186/s12891-015-0664-5

**Published:** 2015-08-27

**Authors:** Iván Prieto-Potin, Raquel Largo, Jorge A Roman-Blas, Gabriel Herrero-Beaumont, David A Walsh

**Affiliations:** Bone and Joint Research Unit, Service of Rheumatology, IIS-Fundación Jiménez Díaz, Autonomous University of Madrid, Avda Reyes Católicos, 2, Madrid, 28040 Spain; Arthritis Research UK Pain Centre, Department of Academic Rheumatology, University of Nottingham, City Hospital, Clinical Sciences Building, Hucknall Road, Nottingham, NG5 1PB UK

## Abstract

**Background:**

Multinucleated giant cells have been noticed in diverse arthritic conditions since their first description in rheumatoid synovium. However, their role in the pathogenesis of osteoarthritis (OA) or rheumatoid arthritis (RA) still remains broadly unknown. We aimed to study the presence and characteristics of multinucleated giant cells (MGC) both in synovium and in subchondral bone tissues of patients with OA or RA.

**Methods:**

Knee synovial and subchondral bone samples were from age-matched patients undergoing total joint replacement for OA or RA, or non-arthritic post mortem (PM) controls. OA synovium was stratified by histological inflammation grade using index tissue sections. Synovitis was assessed by Krenn score. Histological studies employed specific antibodies against macrophage markers or cathepsin K, or TRAP enzymatic assay.

**Results:**

Inflamed OA and RA synovia displayed more multinucleated giant cells than did non-inflamed OA and PM synovia. There was a significant association between MGC numbers and synovitis severity. A TRAP negative/cathepsin K negative Langhans-like subtype was predominant in OA, whereas both Langhans-like and TRAP-positive/cathepsin K-negative foreign-body-like subtypes were most commonly detected in RA. Plasma-like and foam-like subtypes also were observed in OA and RA synovia, and the latter was found surrounding adipocytes. TRAP positive/cathepsin K positive osteoclasts were only identified adjacent to subchondral bone surfaces. TRAP positive osteoclasts were significantly increased in subchondral bone in OA and RA compared to PM controls.

**Conclusions:**

Multinucleated giant cells are associated with synovitis severity, and subchondral osteoclast numbers are increased in OA, as well as in RA. Further research targeting multinucleated giant cells is warranted to elucidate their contributions to the symptoms and joint damage associated with arthritis.

**Electronic supplementary material:**

The online version of this article (doi:10.1186/s12891-015-0664-5) contains supplementary material, which is available to authorized users.

## Background

Osteoarthritis (OA) is a chronic disease affecting the whole joint with progressive changes in the articular cartilage. Changes also in the synovium and subchondral bone are closely associated with symptoms [1], and are thought to contribute to cartilage damage [2]. Rheumatoid arthritis (RA) is characterized by inflammation in the synovium, and also in subchondral bone [3].

Synovitis in RA drives pain, cartilage degradation and pannus formation with subsequent erosions. It is increasingly recognized that synovitis is also observed both in early and in late OA [4]. Indeed, synovitis predicts structural severity and progression of tibiofemoral cartilage damage in OA [5]. Histological features of OA synovitis include synovial lining hyperplasia, infiltration of macrophages and lymphocytes, angiogenesis and fibrosis [6]. OA synovitis may sometimes resemble that which is characteristic of RA. Subchondral inflammation might also contribute to increased bone turnover and joint damage in both OA and RA [7]. Although OA and RA differ in their aetiologies, with mechanical factors being key to knee OA progression and specific immunity driving RA, common mechanisms may contribute to joint damage and pain in both conditions.

Macrophage infiltration is a characteristic feature of synovitis, and is associated with radiographic joint damage both in OA [6] and in RA [8]. Macrophages can fuse to form multinucleated giant cells (MGCs) and this represents a cellular specialization for improved phagocytosis [9]. Although these macrophage polykaryons, generally classified into Langhans (LGC) and foreign body giant cells (FBGC), resemble osteoclasts morphologically, they differ in their functional role. FBGC derived from tissue surrounding total joint arthroplasties were found to express tartrate-resistant acid phosphatase (TRAP) [10]. In fact, many osteoclasts markers, such as TRAP, cathepsin K or metalloproteinase (MMP)−9, among others, can also be expressed by both FBGC and LGC at low levels [11]. In addition, the expression of specific chemokines such as chemokine (C-C motif) ligand (CCL)−2,−3,−4,−5 and−9 have been found to be highly expressed by FBGCs in comparison to osteoclasts [12]. However, whereas osteoclasts are capable of forming lacunar pits of resorption in bone, LGC and FBGC are not [13].

The formation of giant cells in non-skeletal tissues can arise as a result of chronic inflammation due to the presence of foreign material or persistent pathogens. The physiological role of MGCs includes remodelling of granuloma-associated extracellular matrix and clearance of foreign particles from tissues [14]. *In vitro* studies of human tuberculous granulomas have shown a phagocytic activity of LGCs in the presence of low virulence mycobacterium whereas these cells were incapable of phagocytosis in the presence of highly virulent mycobacteria, despite still retaining a strong antigen presentation capability [15]. FBGC together with their macrophage precursors adhere to different synthetic surfaces and degrade or resorb particles that are too large to permit macrophage phagocytosis [16]. Another MGC subtype, foam-like (sometimes called Touton cells), is characterized by lipid uptake [17].

MGCs have been described in inflammatory infiltrates in RA [18], but have also been noticed in the synovium of other arthritic conditions, such as tuberculous arthritis, traumatic arthritis, villonodular synovitis [19], ankylosing spondylitis, psoriasis and OA [20]. Despite the first description of synovial giant cells in RA by Grimley and Sokoloff in 1966 [21], their role in the pathogenesis of OA remains largely unknown. Some recent reports described the presence of these cells in OA synovium [22, 23], whereas others report few or none [24, 25].

Subchondral bone turnover is increased in OA, as evidenced by bone formation and resorption biomarkers [26], as well as by imaging techniques, including radiography [27], computerized tomography, magnetic resonance imaging [28], dual X-ray absorptiometry and scintigraphy [29]. Indeed, recent data from animal models suggest that subchondral bone remodelling might precede and mediate cartilage damage in OA [30], and treatments that target osteoclasts might reduce OA pain [31]. Although activated osteoclasts might be involved in the pathogenesis of OA and RA [32, 33], their relative abundance in the subchondral bone of these diseases remains unknown.

In this study we aimed to study the presence of MGCs in both synovium and subchondral bone tissues of patients with OA and further characterize their phenotypes. We also investigated their relationship with synovial inflammation, and compared our findings with MGCs seen in RA.

## Methods

### Patients

An age and sex matched cross-sectional comparative study was performed on knee samples from people with RA (*n* = 21) or OA (*n* = 42) purposively selected from the Arthritis Research UK Pain Centre Joint Tissue Repository. Informed consent was gained from each donor (for PM cases, next of kin) according to protocols approved by the United Kingdom National Research Ethics Service (Nottingham Research Ethics Committee 1 (05/Q2403/24) and Derby Research Ethics Committee 1 (11/H0405/2)).

All participants in the OA and RA groups satisfied ACR classification criteria for knee OA [34] or RA [35]. RA samples were from consecutive cases collected at joint replacement surgery. Patients with RA were clinically examined prior to joint replacement surgery, a 28-joint Disease Activity Score (DAS28) calculated, and disease activity was classified as high, moderate or low according to European League Against Rheumatism criteria [36]. In order to explore possible associations with synovitis, OA samples, also collected at joint replacement surgery, were purposively selected according to brief histological grading of the severity of synovitis, as previously described [37], and allocated into 2 groups; non-inflamed OA (OANI; *n* = 21) without lining hyperplasia or increased cellularity (grade 0 and 1), or inflamed OA (OAI, *n* = 21) with lining cell hyperplasia and marked hypercellularity (grade 3). The latter group represented 28 % from the screened OA total knee replacement samples. In order to compare MGC phenotypic characteristics between OA and RA, and for correlation analyses, OANI and OAI samples were combined to give single OA group. Non-arthritic post-mortem (PM) cases were used as controls (*n* = 21). PM cases were selected as non-arthritic if there was no reported arthropathy on case note review, and relatives were unaware of the deceased reporting knee pain during the past year, and no Heberden’s nodes or knee joint osteophytes were observed on macroscopic examination post mortem. OA and PM cases were age matched to RA cases by screening consecutive eligible cases for each disease group aged within 2 years of each index RA case.

Synovium and mid-coronal fragments of medial tibial plateaux were collected from selected patients and used for enzymatic histochemistry and immunohistochemistry. Samples were obtained, formalin-fixed, decalcified in 10 % ethylenediaminetetraacetic acid (tibial plateaux only) and paraffin embedded at Sherwood Forest Hospitals NHS Foundation Trust, Sutton in Ashfield, UK.

### Histopathology

All histological assessments were undertaken by a single researcher (IP-P) who was blinded to diagnostic group. Synovitis severity was firstly graded according to Haywood et al., 2003, as criteria for inclusion of patients in both OANI and OAI groups. This score, mainly based in lining hyperplasia grading, was complemented by using the Krenn score which provides more detailed characterization of synovial inflammation using haematoxylin and eosin stained sections, as previously described in both OA and RA [38]. Briefly, in addition to lining hyperplasia, fibrovascular alterations in the interstitium and cellular infiltration were evaluated using 0–3 point subscales, where 0 indicates absence, 1 mild, 2 intermediate and 3 strong. The total score was obtained from the sum of partial grades with a maximum total score of nine. Spearman’s rank correlation coefficients were used to explore association between the two methods for scoring synovitis in OA.

Synovial MGCs were identified as large cells showing three or more nuclei inside their cytoplasm in haematoxylin and eosin stained sections, thereby avoiding confounding by mitotic cells with two nuclei. Classification of different morphological variants was performed according to the arrangement and composition of their organelles. Foreign body giant cells were identified as MGCs with many nuclei diffusely distributed throughout the cytoplasm [16]. Langhans giant cells, in contrast, were recognized by the multiple nuclei arranged in a horseshoe shape [9]. Multinucleated foam giant cells were characterized by numerous nuclei clustered together, surrounded by a foamy cytoplasm [17]. Although multinucleated plasma cells are not derived from macrophage lineage, we also assessed these subtype of MGCs by their eccentric nuclei with a dense distribution of chromatin [39]. Osteoclasts were sought in synovium as TRAP-positive MGCs, and also identified in subchondral bone as TRAP-positive MGCs adjacent to bone or calcified cartilage.

Sections were analyzed through 10× and 40× objective lenses to identify MGC location within the lining and sublining layers and to assess cell morphology, respectively. An image of each MGC was captured using a 40× objective (AxioCam, Carl Zeiss Ltd., North Ryde, NSW, Australia) and maximum diameter (μm) was measured with AxioVision Rel 4.8 software (Carl Zeiss Ltd., Welwyn Garden City, UK). Sections of synovium were scanned with EPSON Expression XL 10000 scanner (Epson America Inc, Long beach, CA, USA) for measuring the synovial biopsy area with AxioVision Rel 4.8 software (Carl Zeiss Ltd., Welwyn Garden City, UK). MGC densities are expressed as number per mm^2^.

### TRAP enzymatic activity assay and immunohistochemistry

Synovial and tibial plateaux samples were serially sectioned at 5 μm, dewaxed, rehydrated and incubated overnight in 1 mM CaCl_2_ and 1 mM MgCl_2_, then stained for TRAP, according to manufacturer’s protocol (Sigma, St Louis, Mo, USA). Mid-coronal fragments of medial tibial plateaux were divided into three equal parts (external, central and internal) and TRAP positive cells with multiple nuclei were counted in each part as positive MGCs under light Axioskop-50 microscope (Carl Zeiss Ltd., Welwyn Garden City, UK), using a 40× objective lens. Total osteoclastic activity was expressed as the sum of TRAP positive cells of each part per mm^2^.

Macrophages were visualized using mouse monoclonal anti-CD68 primary antibody, 1 mg/ml at a 1/200 dilution (Dako, clone PG-M1, M0876, Golstrup, Denmark) and a nickel-enhanced ABC-peroxidase technique [40] (Vector laboratories, CA, USA). Cathepsin K positive cells were visualized with a mouse monoclonal anti-cathepsin K (Abcam, ab66237, Cambridge, UK) antibody, 0,5 mg/ml at a 1/100 dilution, and ABC-peroxidase developed with diaminobenzidine. Horse anti-mouse IgG secondary antibodies, 0,5 mg/ml (Vector laboratories, BA2001, CA, USA), were used at dilutions 1/100 for CD68 and 1/4000 for cathepsin K. Antigen retrievals used were 1 mg/ml pepsin in 0.5 M acetic acid, 2 h at 37 °C for CD68, and 10 mM citrate buffer, pH 6, 30 min at 85 °C for cathepsin K. Sections were counterstained with eosin for CD68 and with haematoxylin for cathepsin K; and mounted with di-n-butylphthalate in xylene (DPX mounting medium, VWR International Ltd., Lutterworth, UK).

### Statistical analysis

All statistical analyses were performed using SPSS version 17.0 software for Windows (IBM, New York, NY, USA). Descriptive data are expressed as the median ± interquartile range (IQR). Proportions were compared using Chi-squared test. Associations are expressed using Spearman’s rho correlation coefficient. Comparisons between multiple groups used Kruskal-Wallis tests with Bonferroni correction of post hoc Mann–Whitney U tests. *P* < 0.05 was considered significant.

## Results

### Demographic and clinical details

Table 1 shows the demographic and clinical characteristics of participants. Age matching was successful, and there were no significant gender differences between groups. Glucocorticoids had been received by six participants (3 PM and 3 RA), and bisphosphonates by 2 RA, one also receiving calcium and vitamin D supplementation. Non-steroidal anti-inflammatory drugs were used in analgesic doses by 16 participants (6 OANI, 4 OAI and 6 RA). Disease modifying anti-rheumatic drugs (DMARDs) were used by 17 participants with RA (11 patients were using methotrexate, 5 sulphasalazine and 1 ciclosporin A). Five patients used a combination of 2 DMARDs, including hydroxychloroquine, rituximab or etanercept). DAS28 score showed five patients with high disease activity, eight with moderate and eight with low activity.

**Table 1:** Demographic and clinical details

	PM	OANI	OAI	RA
N	21	21	21	21
Age, years	68 (63–73)	68 (63–72)	68 (63–72)	68 (63–72)
Gender, female, %	43	48	38	50
ESR, mm/hr	Not available	7 (5–13)	14 (7–22)	22(13–34)*
Serum CRP, mg/L	Not available	5(2–5)	5(5–6)	12(5–47)*^#^
DAS28	−	−	−	3,5 (2,4–4,8)

Data were available for erythrocyte sedimentation rate (ESR) on 13 non-inflamed OA (OANI), 18 inflamed OA (OAI) and 20 rheumatoid arthritis (RA) cases; and for serum C-reactive protein (CRP) on 12 OANI, 18 OAI and 21 RA cases. DAS28; 28 joint Disease Activity Score. Values represent medians and IQR. *p < 0.01 vs. OANI; ^#^ *p* < 0.01 vs. OAI

### Characterization of synovial MGC in OA and RA

**Fig. 1:**
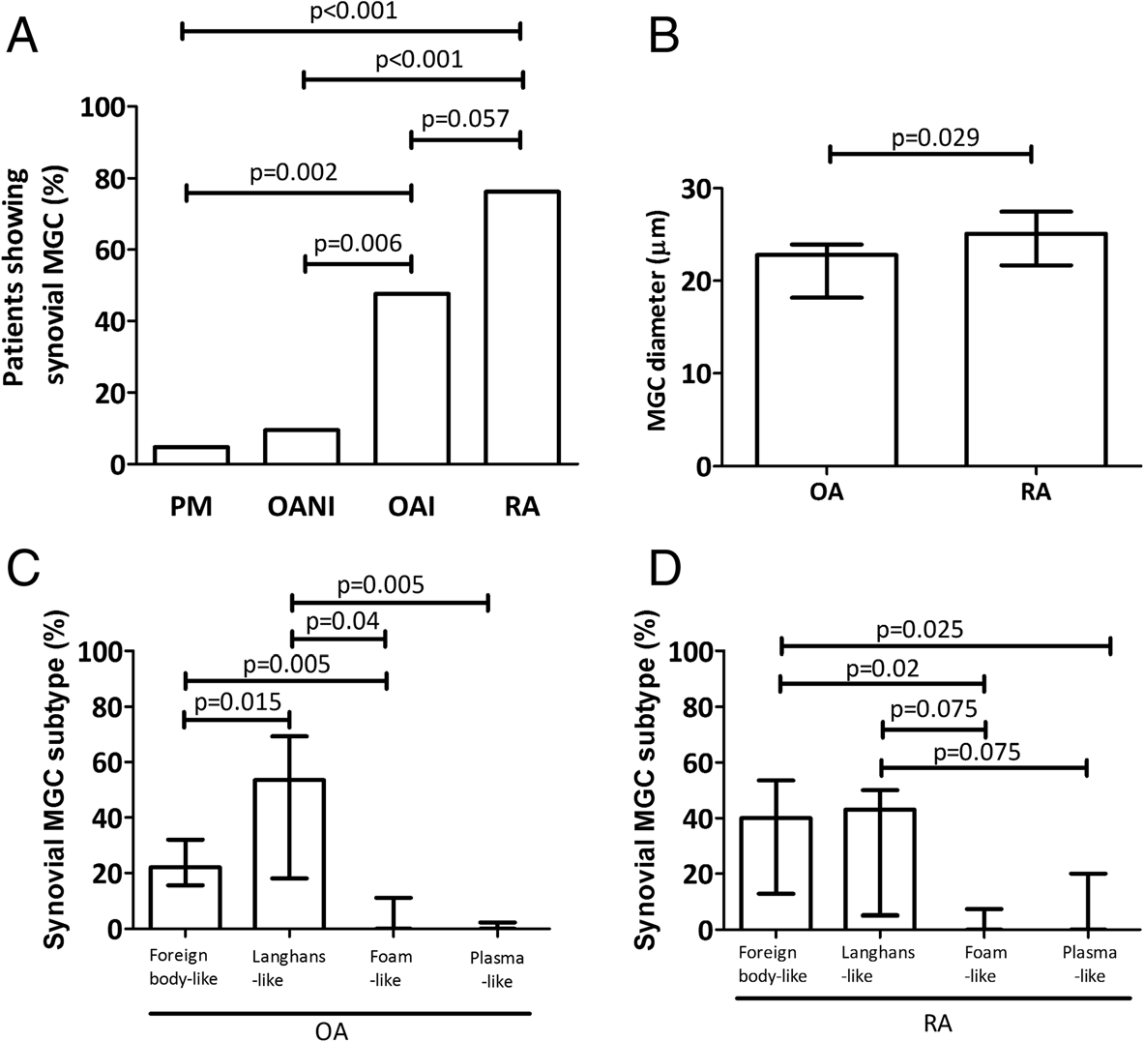
Characterization of MGC subtypes in OA and in RA. **a**. A greater proportion of inflamed OA (OAI) and RA cases displayed MGCs than did non-inflamed OA (OANI) or non-arthritic (post mortem, PM) controls. **b**. Increased MGC size in RA compared with OA cases. **c** and **d**. Relative abundance of morphological MGC subtypes observed in OA (**c**) and RA (**d**) synovia. Bars in **b**-**d** represent medians, with IQRs indicating between MGC heterogeneity

**Fig. 2:**
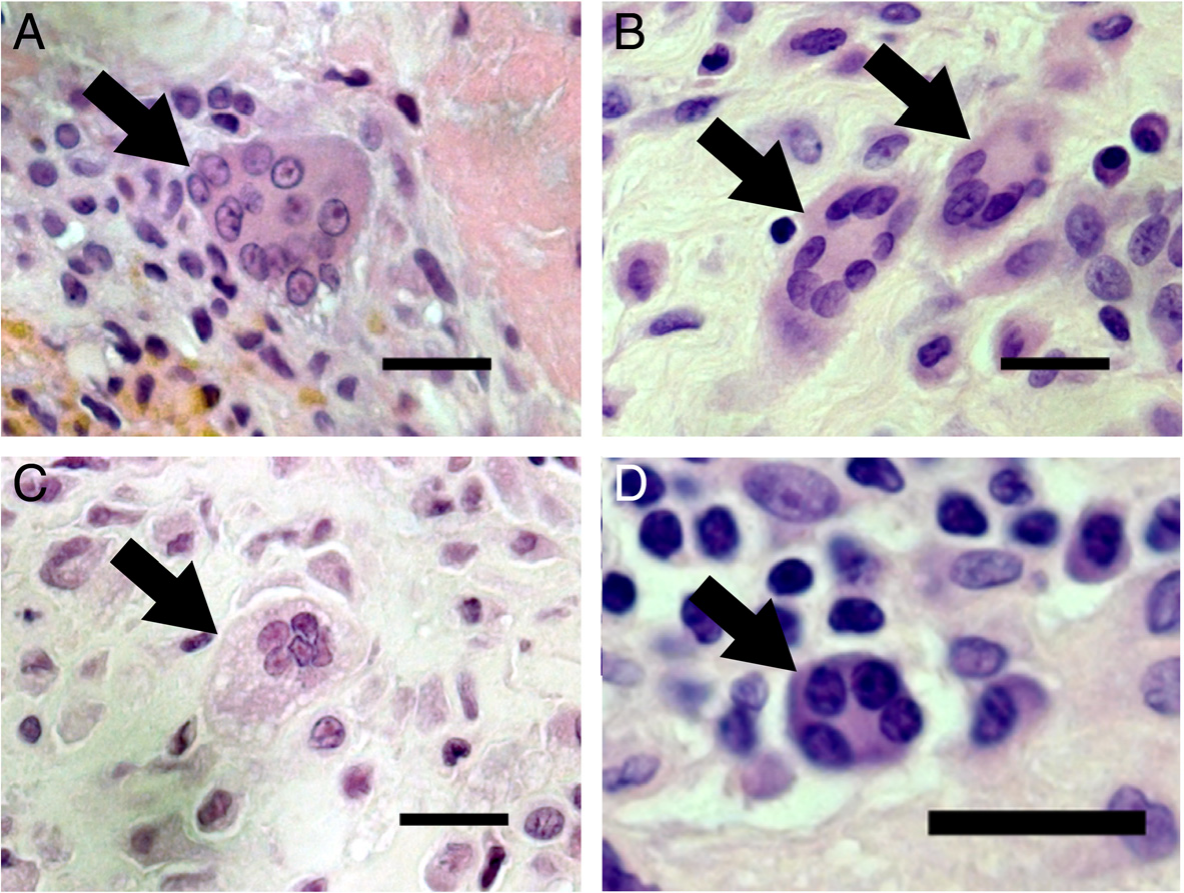
Morphological characterization of synovial MGC subtypes in OA and in RA. Foreign body giant cells were identified as MGCs with many nuclei diffusely distributed throughout the cytoplasm (**a**). Langhans giant cells were recognized by the multiple nuclei arranged in a horseshoe shape (**b**). Foam MGC subtype was characterized by numerous nuclei clustered together, surrounded by a foamy cytoplasm (**c**). Plasma MGC subtype was assessed by their eccentric nuclei with a dense distribution of chromatin (**d**). Overall MGC subtypes were identified in either OA or RA. Arrows indicate each different MGC subtype in synovium. Haematoxylin and eosin staining. Scale bars = 20 μm

The percentage of patients who displayed MGCs in synovia was higher in RA and OAI when compared with OANI and PM (*p* < 0.01, Fig. 1a). MGCs were observed in more OAI than OANI synovia, *p* = 0.006, both in lining and sublining synovial layers. Absolute MGC number adjusted for synovial section area (median (IQR)) was significantly lower in OA (0 (0–0.06) per mm^2^) than in RA (0.10 (0.01–0.22) per mm^2^, *p* = 0.001). In OA synovia, MGCs were smaller than those observed in RA (Fig. 1b). The full range of different MGC subtypes identified by their nuclear organization was observed in each disease (Fig. 2). However, whereas Langhans-like MGCs were the most abundant subtype in OA (Fig. 1c), foreign body-like, in addition to Langhans-like MGCs, were prominent in RA synovia (Fig. 1d). Foam-like and plasma-like MGCs were less abundant in both disease groups. Foam-like MGCs were identified near to and surrounding fat cells in inflamed synovia from patients with either OA or RA. Furthermore, multiple mononuclear CD68 positive cells were found in a crown-like structure encircling adipocyte cells (Additional file 1).

**Fig. 3:**
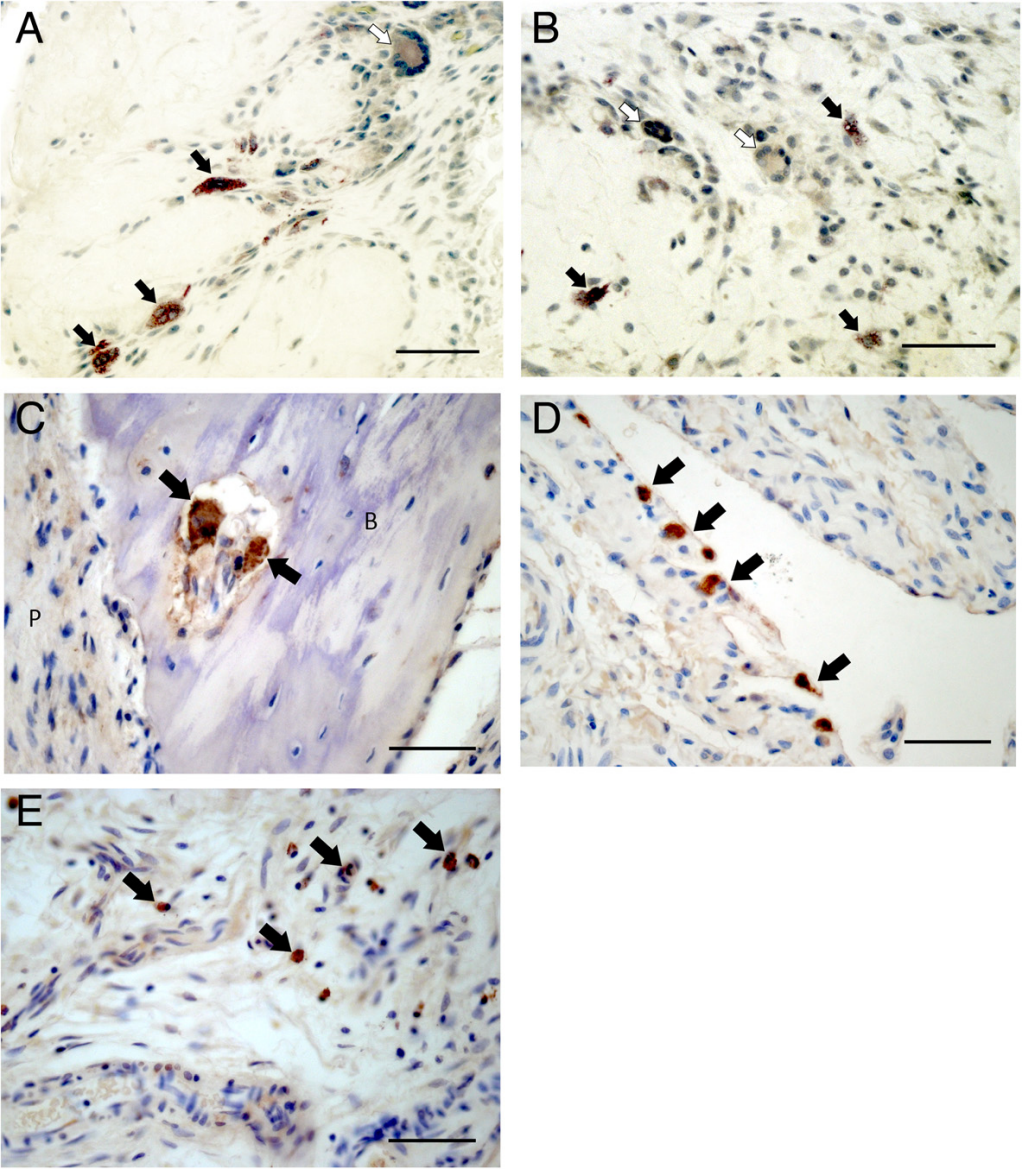
TRAP and cathepsin K enzyme activity in synovium from OA and RA patients. **a** and **b**. Representative synovial TRAP-positive and TRAP-negative MGCs both in inflamed OAI (**a**) and RA (**b**) cases. **c**. Cathepsin K-immunoreactive MGC localized to synovial pannus adjacent to bone surface in RA and OA tibial plateaux. **d** and **e**. Cathepsin K-immunoreactive mononuclear synovial lining (**d**) and sublining (**e**) cells in OAI and in RA synovium. Filled arrows indicate TRAP-positive or cathepsin K-positive cells. Open arrows indicate TRAP-negative MGCs. *P* = pannus and *B* = bone, scale bars = 50 μm

We also analyzed TRAP activity and Cathepsin K immunoreactivity as markers of MGCs in synovium [41]. We found that most of the foreign body-like MGCs positively stained for TRAP, whereas many MGCs displaying characteristics of Langhans, multinucleated foam-like and plasma cells were TRAP negative (Figs. 3a and b). Cathepsin K-immunoreactive MGCs were only localized to synovial pannus adjacent to bone surfaces in RA tibial plateau (Fig. 3c), and were not observed in synovial tissue distant from bone or cartilage. Some mononuclear synovial lining and sublining cells also stained positive for cathepsin K, both in OAI and in RA synovia (Figs. 3d and e).

### Synovial inflammation in OA and RA

**Fig. 4:**
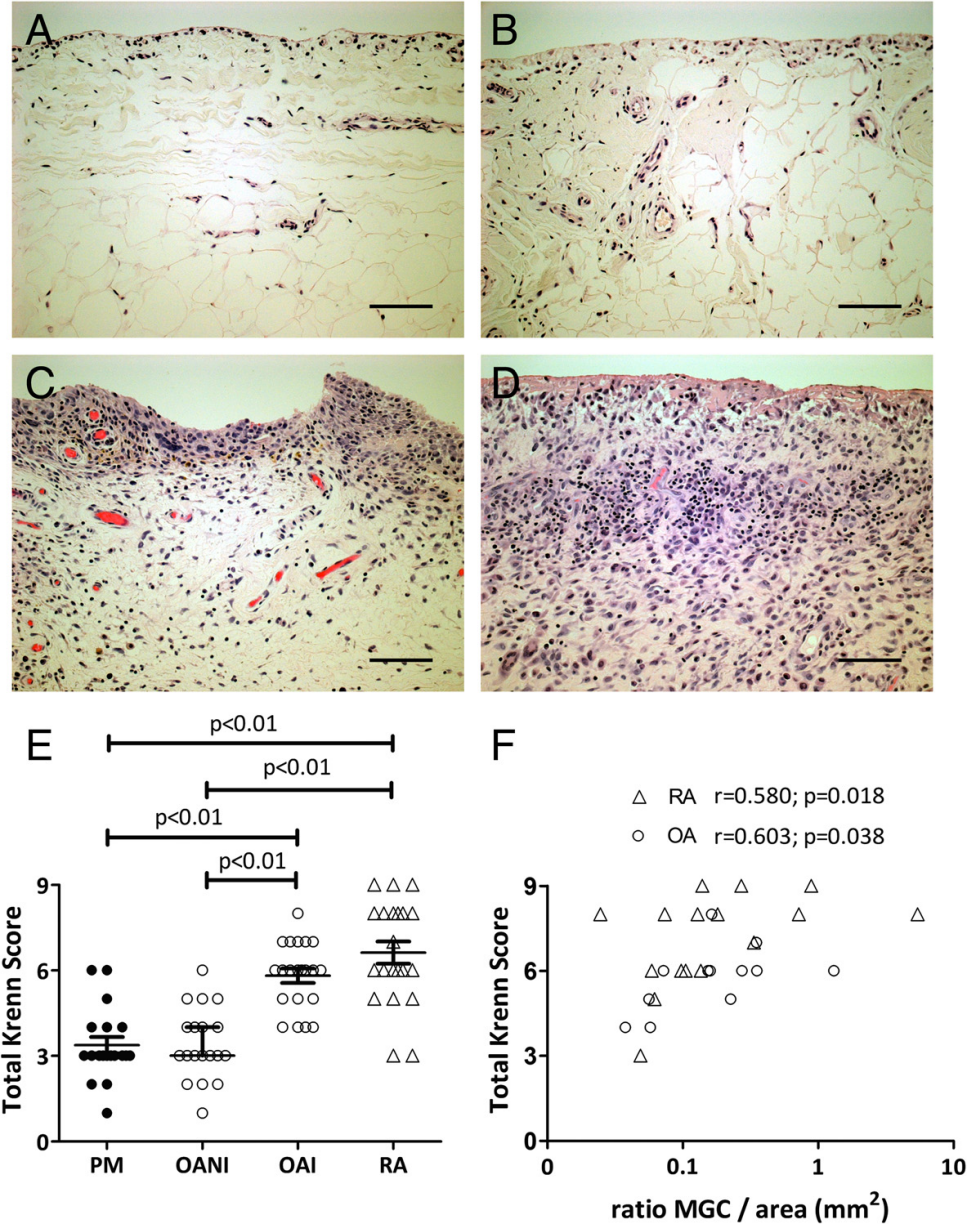
Synovial inflammation in OA and RA. **a**-**d**. Representative sections of synovia from cases with median Krenn scores for each disease group stained with haematoxylin and eosin. Panels represent post-mortem, PM (**a**), non-inflamed OA, OANI (**b**), inflamed OA, OAI (**c**) and RA (**d**) cases. Scale bars = 100 μm. **e**. Synovitis, determined as total Krenn score was greatest in OAI and RA cases. **f**. Numbers of MGCs per area of synovium were positively correlated with synovitis scores both in OA and in RA cases

Haywood synovitis score was significantly correlated with Krenn score in patients with OA (*r* = 0791; *p* < 0001). OAI and RA samples were each characterized by synovial lining hyperplasia, sublining fibrosis and stromal vascularization, along with inflammatory infiltrates often containing follicle-like lymphoid aggregates (Figs. 4a-d). OAI and RA synovia had higher synovitis grades than OANI and PM (each *p* < 0.001, Fig. 4e). Scores for each Krenn subscale (stromal cell density, lining hyperplasia and inflammatory infiltrate) were increased in OAI and RA synovia in comparison with OANI and PM (each *p* < 0.05).

**Fig. 5:**
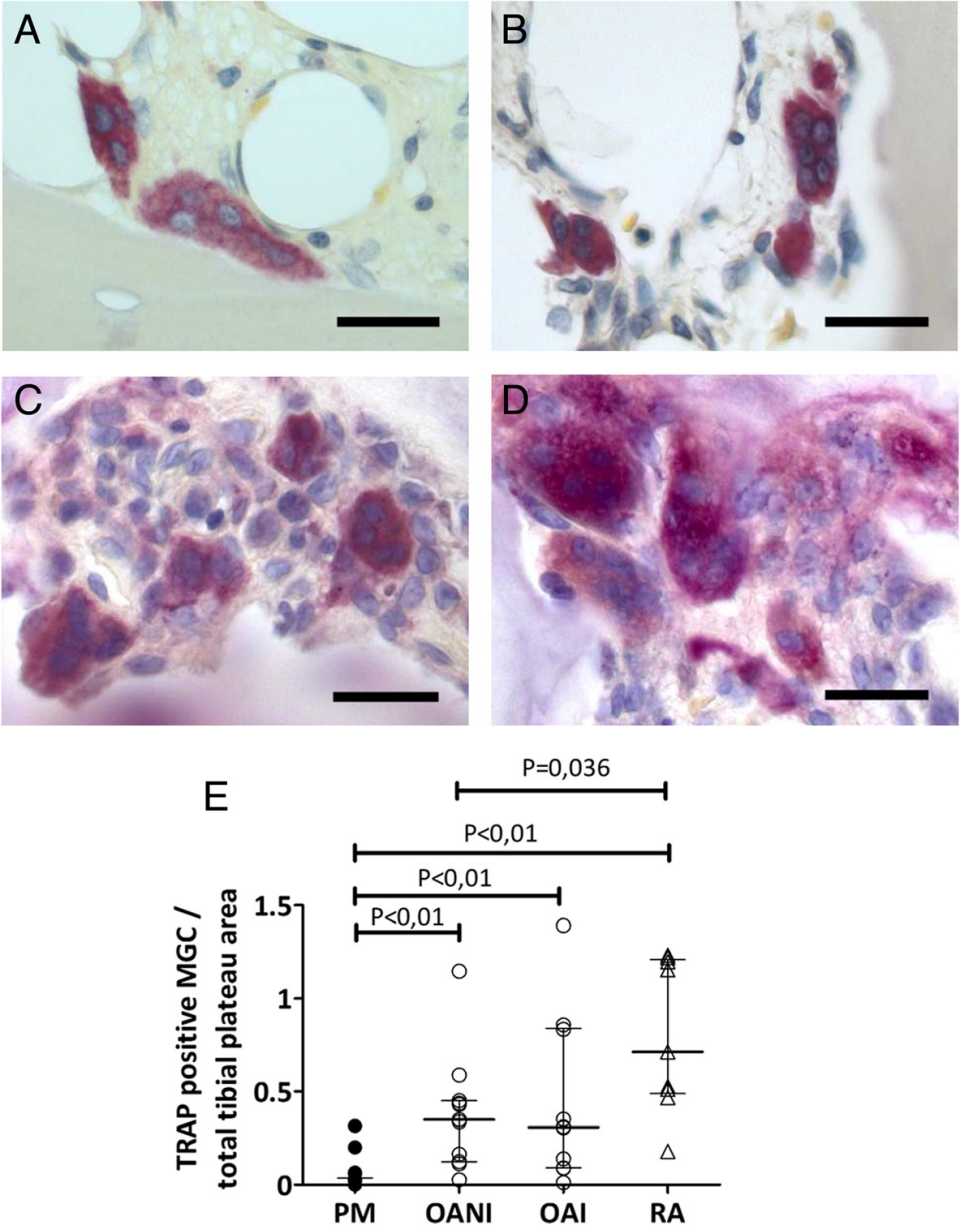
Subchondral bone TRAP positive osteoclast in OA and in RA. **a**-**d**. Representative sections from non-arthritic patients (**a**), with OANI (**b**), OAI (**c**) and RA (**d**) showing TRAP positive osteoclasts adjacent to bone surfaces. Scale bars = 20 μm. **e**. **c**. Absolute TRAP-positive osteoclast number adjusted for the area of mid-coronal tibial plateau fragments. Bars represent medians with IQRs

Synovitis grade and synovial MGC density were significantly associated, both in OA and RA groups (Fig. 4f). Furthermore, MGC densities were higher in cases with greater inflammatory infiltration in OA (*r* = 0.63; *p* = 0.03), and with higher stromal cellularity in RA (*r* = 0.53; *p* = 0.03). Associations between serum CRP levels and MGC density did not reach statistical significance in either OA or RA (*r* = 0.30; *p* = 0.38 and *r* = 0.09; *p* = 0.75 respectively). Likewise, serum ESR was not significantly associated with MGC density in OA or RA (*r* = −0.20; *p* = 0.55 and *r* = −0.12; *p* = 0.68, respectively).

### Subchondral bone osteoclasts in OA and RA

We then analyzed the presence of multinucleated cells in subchondral bone of medial tibial plateaux. MGCs adjacent to bone surfaces that displayed TRAP activity and a random nuclear organization were counted as osteoclasts. Increased numbers of TRAP-positive osteoclasts were observed in subchondral bone from people with RA, or with OA and either non inflamed or inflamed synovium (Figs. 5b-d) compared with non-arthritic PM controls, *P* < 0.05 in all comparisons (Fig. 5a). No significant differences in osteoclast numbers were found between OAI and RA groups, whereas TRAP-positive subchondral osteoclasts were more abundant in RA compared to the non-inflamed OA group, *p* = 0.036 (Fig. 5e). Associations between subchondral TRAP-positive osteoclasts and synovial MGC numbers were not statistically significant in either OA (*r* = 0.30, *P* = 0.62) or RA (*r* = −0.14; *P* = 0.76).

## Discussion

In this study we have shown that MGCs were increased in OA, especially in inflamed synovia which presented similar MGC numbers to those observed in RA. MGC subtypes might differ between OA and RA, with a relative sparsity of foreign body MGCs in OA. In addition, we provide histopathological evidence of increased subchondral osteoclastic activity in OA, as well as in RA. Together, our findings indicate a clear activation of mononuclear phagocyte system cells, both in synovium and in subchondral bone, and both in OA and in RA.

Synovial inflammation is a common feature of OA, although the intensity and nature of inflammation may differ between OA and RA [42]. It is currently unknown whether synovitis characterizes a subgroup of people with OA, or may occur during phases of the disease in all patients. In cross sectional studies, the extent of synovitis varies between cases, ranging from normal to severe inflammation [43]. Cases purposively selected for the current study represented both ends of this spectrum in order to explore associations between MGCs and synovitis, and to characterize MGCs in detail. The inflamed OA group studied here, comprised 28 % of consecutive cases donating tissue at joint replacement surgery for OA, and therefore represents a common inflammatory phenotype amongst people with end stage OA. A similar prevalence of synovitis was observed in a previous study of 104 participants undergoing joint replacement surgery for knee OA [37].

We have also demonstrated that there is a prevalent subgroup of people with OA in whom the numbers of MGCs are comparable to those in people with RA at the time of joint replacement surgery. Furthermore, MGC densities were significantly associated with the total synovitis score indicating that this is a cellular characteristic of synovitis that is shared between OA and RA, suggesting shared inflammatory mechanisms. Varying estimates of the prevalence of MGCs in synovium from people with OA or RA might reflect the degree of synovitis in cases selected for study.

In the current study, statistically significant associations were not detected between ESR or serum CRP and MGC numbers, suggesting local co-ordination of MGCs and inflammation within the synovium. However, larger populations would need to be investigated to explore smaller possible systemic influences on MGC formation. A limitation of cross sectional studies such as ours is that differences between groups may be caused by unmeasured confounding factors. We have attempted to minimize possible confounding by carefully matching cases by gender and age and although our assessments were undertaken blinded to diagnostic group, we cannot be certain that detection of differences between groups was entirely free from observer bias. Participants with RA were more likely to be using glucocorticoids, bisphosphonates or DMARDs than were those with OA, and inhibition of MGCs by anti-inflammatory drugs might have led us to underestimate the magnitude of increased MGC and osteoclast numbers in RA.

Our data extend those from a previous study showing two main types of giant cells in arthritic synovium [44]. We have demonstrated that OA and RA synovia exhibit distinct patterns of MGC subtype frequency, Langhans-like MGC being the predominant subtype in people with OA. Langhans-like MGCs contribute to processing and presentation of antigens and the development of specific immune responses, and their presence in OA might indicate a contribution of specific immunity to OA [45–47]. The pathological relevance of smaller MGC size and lower prevalence of foreign body like giant cells in OA than in RA remains unclear, but we speculate that deficiencies in processing cartilage degradation products might contribute to synovitis in OA.

In addition to the main Langhans and foreign-body MGC subtypes, we also observed foam-like MGCs in synovia from people undergoing joint replacement surgery for either OA or RA. Previous studies have most frequently localized foam-like MGCs in lesions containing cholesterol and lipid deposits, such as xanthomas or xanthogranulomas [48], and these MGCs might develop when a cell fusion stimulus is also accompanied by a factor stimulating lipid uptake [49]. The contribution of foam-like MGCs to synovitis in OA and RA deserves further study, given recognized associations between human arthritis, obesity and metabolic syndrome [50], and preclinical evidence that synovial foam-like MGCs might be associated with increased bone resorption [51].

We found that foreign body-like MGCs positively stained for TRAP, indicating that these cells might have osteoclastic activity [52], whereas Langhans MGCs were negative for TRAP. Absence of TRAP activity in Langhans MGCs has also been noted in canine and feline neoplasia [53]. Cathepsin K was not a specific marker of MGC in inflamed synovium, where it was also localized to mononuclear cells more distant from bone and cartilage. We did not identify cathepsin K activity in MGCs that were distant from bone or cartilage in human synovia, in contrast to previous suggestions that cathepsin K-positive staining might be a general feature of MGCs [41]. Cathepsin K-negative MGCs have also been reported in human breast carcinoma and lung biopsies [54]. Our findings suggest that cathepsin K-immunoreative MGCs in human synovium might represent an osteoclastic MGC subtype. However, we cannot be certain whether TRAP and cathepsin K positive MGCs detected in the synovium were osteoclasts, or alternatively osteoclast precursors. Diverse MGC subtypes have been described previously in various autoimmune, infectious or sarcoid granulomatous diseases. The demonstration of diverse morphological subtypes [14], distribution and immunohistochemical characteristics [55] might reflect discrete functional states of MGCs.

TRAP and cathepsin K positive osteoclasts were localised adjacent to bone and cartilage, both in synovium and in subchondral bone. The formation of resorption subchondral pits by osteoclasts [56] and cells that resorb mineralized and unmineralized articular cartilage [57] contribute to hard tissue remodeling in OA. Increased subchondral bone turnover and resorption might contribute to OA development, and might be features of both early and end stage disease [58]. Our findings suggest a contribution of increased osteoclast activation to increased subchondral bone remodelling both in RA and in OA. Increased numbers of TRAP-positive MGCs in OA subchondral bone reflect augmented osteoclastic activity in animal models of OA [59], and our findings suggest similarly increased subchondral osteoclastic activity in human knee OA. Future studies should explore possible associations between subchondral osteoclast numbers and biochemical or imaging markers of subchondral bone turnover. Subchondral osteoclast numbers were increased in both inflamed and non-inflamed OA subgroups, whereas synovial MGCs were more frequently observed in the inflamed subgroup and were associated with synovitis grade. Furthermore, no statistically significant association was found between the increased numbers of subchondral osteoclasts and synovial MGCs. These data lead us to suggest that synovial inflammation and subchondral bone remodelling each may be regulated by different mediators.

## Conclusion

We found that MGCs were increased in OA, especially in inflamed OA which presented similar MGC numbers to those observed in RA. Synovial MGCs displayed a range of morphological subtypes, with Langhans giant cells being the predominant variant in OA, and both Langhans and foreign body like subtypes in RA. MGCs might contribute to catabolic activity and be regulated by local factors within the synovium, whereas MGCs in subchondral bone might predominantly represent osteoclasts and be regulated by factors that are independent of synovitis. Preclinical studies and early clinical trials support the targeting of osteoclasts in the treatment of OA [31]. Further research is required to determine the potential of targeting MGCs to improve pain and joint damage both in OA and in RA.

## Additional file

Additional file 1:
**Morphological MGC foam subtype and CD68 positive cells surrounding fat cells in OA and in RA.** A and B. MGCs displaying a foam-like subtype were identified near to and surrounding fat cells in inflamed synovia from patients with either OA (A) or RA (B). Haematoxylin and eosin staining. Scale bar = 20 μm. Open arrows indicate foam-like MGC and A = adipocyte. C and D. Multiple mononuclear CD68 positive cells were found in a crown-like structure encircling adipocyte cells in both OA (C) and RA (D). Immunohistochemistry for CD68, using eosin contrast. Scale bar = 100 μm. (TIFF 3238 kb)

## Abbreviations

ACR: American college of rheumatology
CRP: C reactive protein
DMARDs: Disease modifying anti-rheumatic drugs
ESR: Erythrocyte sedimentation rate
FBGC: Foreign body giant cell
IQR: Interquartile range
LGC: Langhans giant cell
MGC: Multinucleated giant cell
OA: Osteoarthritis
OAI: Inflamed osteoarthritis
OANI: Non-inflamed Osteoarthritis
PM: Post-mortem
RA: Rheumatoid arthritis
