# Supplementary figures and images for: Characterization of multinucleated giant cells in synovium and subchondral bone in knee osteoarthritis and rheumatoid arthritis
Source: BMC Musculoskelet Disord. 2015 Aug 27;16:226. doi: 10.1186/s12891-015-0664-5 (PMC4550054; doi:10.1186/s12891-015-0664-5)

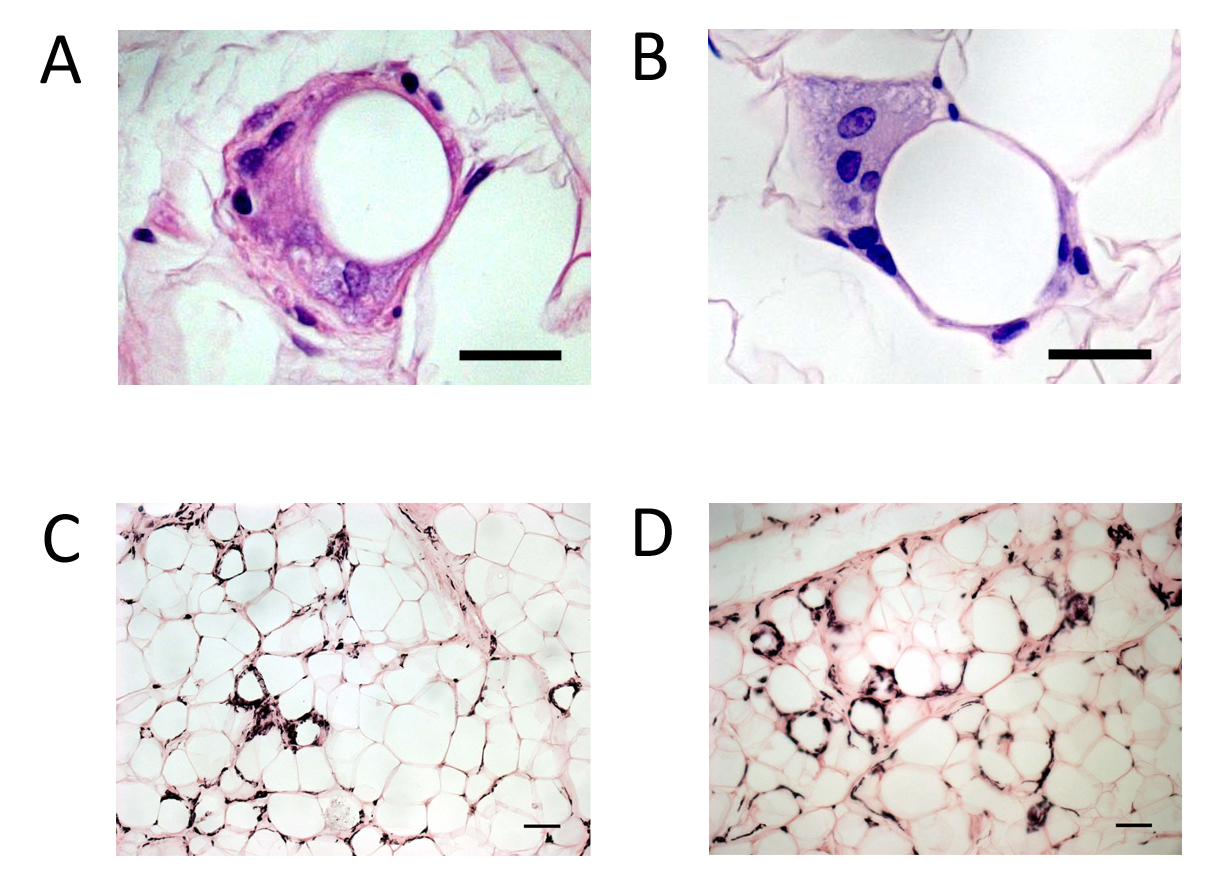

Supplement: Additional file 1: — Morphological MGC foam subtype and CD68 positive cells surrounding fat cells in OA and in RA. A and B. MGCs displaying a foam-like subtype were identified near to and surrounding fat cells in inflamed synovia from patients with either OA (A) or RA (B). Haematoxylin and eosin staining. Scale bar = 20 μm. Open arrows indicate foam-like MGC and A = adipocyte. C and D. Multiple mononuclear CD68 positive cells were found in a crown-like structure encircling adipocyte cells in both OA (C) and RA (D). Immunohistochemistry for CD68, using eosin contrast. Scale bar = 100 μm. (TIFF 3238 kb) [file 12891_2015_664_MOESM1_ESM.tiff]
